# Successful Organizational Strategies to Sustain Use of A-CHESS: A Mobile Intervention for Individuals With Alcohol Use Disorders

**DOI:** 10.2196/jmir.3965

**Published:** 2015-08-18

**Authors:** James H Ford II, Esra Alagoz, Susan Dinauer, Kimberly A Johnson, Klaren Pe-Romashko, David H Gustafson

**Affiliations:** ^1^ University of Wisconsin - Madison Center for Health Systems Research and Analysis Madison, WI United States; ^2^ University of Wisconsin - Madison Center for Health Enhancement Systems Studies Madison, WI United States

**Keywords:** mHealth, substance abuse disorder, sustainability, funding, engagement, staff, client, implementation

## Abstract

**Background:**

Mobile health (mHealth) services are growing in importance in health care research with the advancement of wireless networks, tablets, and mobile phone technologies. These technologies offer a wide range of applications that cover the spectrum of health care delivery. Although preliminary experiments in mHealth demonstrate promising results, more robust real-world evidence is needed for widespread adoption and sustainment of these technologies.

**Objective:**

Our aim was to identify the problems/challenges associated with sustained use of an mHealth addiction recovery support app and to determine strategies used by agencies that successfully sustained client use of A-CHESS.

**Methods:**

Qualitative inquiry assessed staff perceptions about organizational attributes and strategies associated with sustained use of the mobile app, A-CHESS. A total of 73 interviews of clinicians and administrators were conducted. The initial interviews (n=36) occurred at the implementation of A-CHESS. Follow-up interviews (n=37) occurred approximately 12 and 24 months later. A coding scheme was developed and Multiuser NVivo was used to manage and analyze the blinded interview data.

**Results:**

Successful strategies used by treatment providers to sustain A-CHESS included (1) strong leadership support, (2) use of client feedback reports to follow up on non-engaged clients, (3) identify passionate staff and incorporate A-CHESS discussions in weekly meetings, (4) develop A-CHESS guidelines related to client use, (5) establish internal work groups to engage clients, and (6) establish a financial strategy to sustain A-CHESS use. The study also identified attributes of A-CHESS that enhanced as well as inhibited its sustainability.

**Conclusions:**

Mobile apps can play an important role in health care delivery. However, providers will need to develop strategies for engaging both staff and patients in ongoing use of the apps. They will also need to rework business processes to accommodate the changes in communication frequency and style, learn to use app data for decision making, and identify financing mechanisms for supporting these changes.

## Introduction

Emerging as a subsegment of eHealth technologies, mobile health (mHealth) services are growing in importance in health care with the advancement of wireless networks, tablets, and mobile phone technologies [1]. mHealth technologies offer a wide range of applications that cover the spectrum of health care delivery from measurement and diagnostics to treatment and recovery support. A 2013 Pew report states that 56% of the US population owned a smartphone in 2013, which is a 10% increase from the previous year [2]. The possibilities for use of mHealth technology to address care delivery, patient monitoring, and adherence support have created substantial enthusiasm among developers, providers, policy makers, and researchers to use mHealth tools [3]. The Global Mobile Health Market report proposes that one-third of the smartphone users will use some kind of health care app in 2015 [4]. Among the 1980 available therapeutic apps, chronic disease management is the most common type of app [5]. Addiction recovery support apps fall into this category, with substance use disorders for some people being categorized as a chronic condition [6,7]. These apps offer personalized recovery monitoring and easy access to online support communities [8-16]. While some apps rely on text messaging to monitor alcohol and drug use and send reminders to users as an intervention [17,18], more current comprehensive recovery management systems that integrate mobile Internet capabilities and sensors like global positioning satellites (GPS) and accelerometers have also been introduced [9,19]. With personalized systems tailored specifically for user needs and 24/7 availability to patients and caregivers, comprehensive recovery management systems have the potential to take mHealth to a new level if they are well developed, adopted, and used by clients and supported by providers.

Although preliminary experiments in mHealth demonstrate promising results in data collection for health care research [20], medical and health care education [21], remote health care assistance in developing nations [22], and improved treatment outcomes [19], robust real-world evidence is needed for widespread adoption and sustainment of these technologies [23,24]. Drawbacks such as counselor resistance and perceptions of increased staff workload, as well as the cost of owning a smartphone or implementation of the technology, impede the widespread utilization of mHealth technologies in a sustainable manner [25,26].

The purpose of this research was to study the sustainability of the implementation of an mHealth app (A-CHESS) designed to support addiction recovery. A-CHESS is a mobile phone app designed to help people maintain recovery and prevent relapse from drug or alcohol addiction. In this study, the app was provided to the individual by their treatment agency and included ongoing online and sometimes in-person support from agency counselors. At a minimum, agencies provided connection to a 24-hour crisis line within the A-CHESS app for after-hours coverage.

A-CHESS is consistent with self-determination theory and Marlatt’s stages-of-relapse model. The various A-CHESS services align with the elements of self-determination theory: competence, relatedness, and autonomy [27,28]. The app also incorporates a weekly check-in assessment using a version of the Brief Alcohol Monitor (BAM) survey to obtain patient data on recent alcohol and other drug use as well as status on protective and risk factors [29]. A-CHESS uses the BAM survey information to provide feedback for the individual and includes a Bayesian predictive model that estimates the likelihood that the patient will relapse in the coming weeks [30]. Agency counselors are able to see the results of the surveys if the user agrees to share that information.

**Figure 1:**
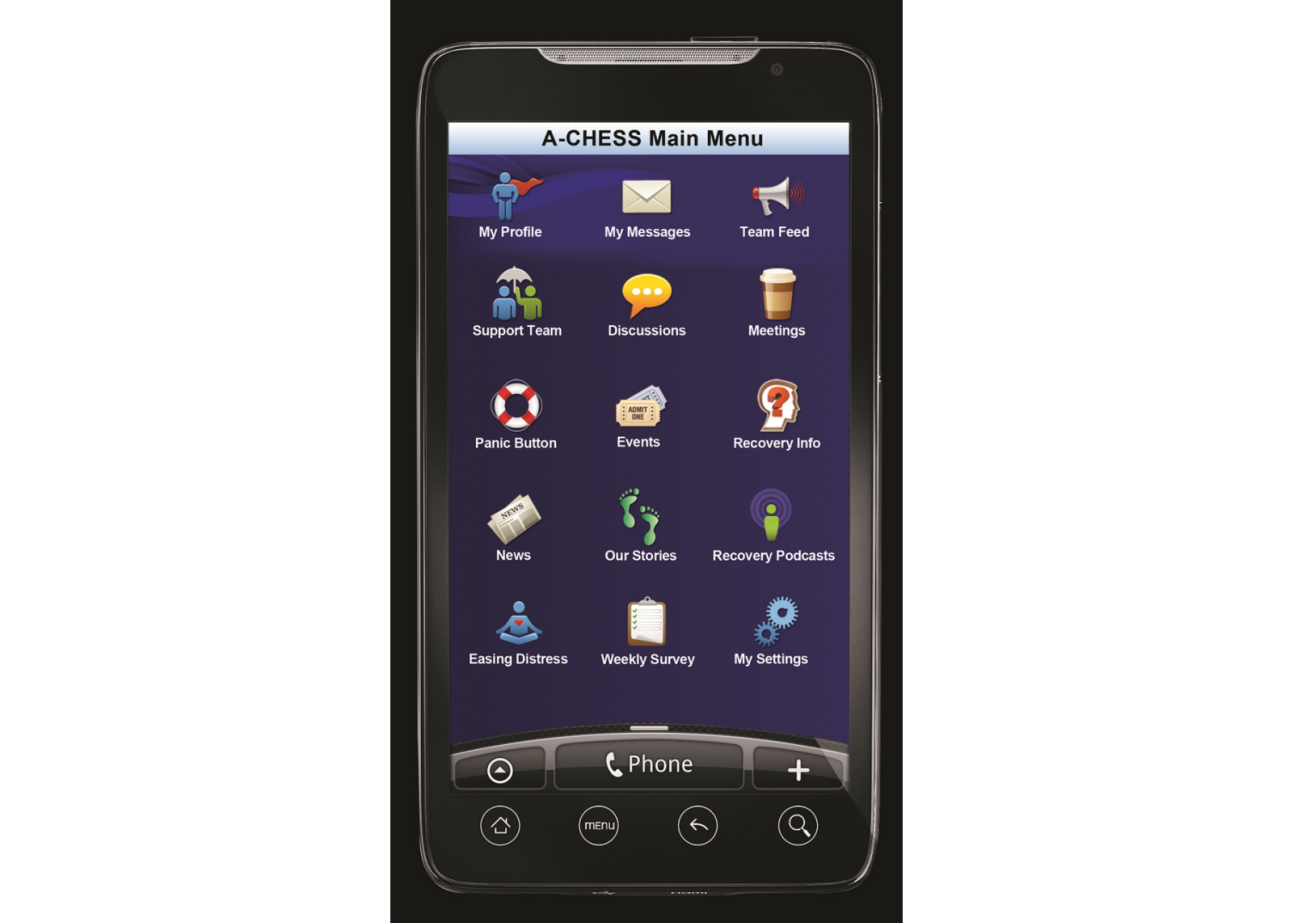
A-CHESS main menu.

**Figure 2:**
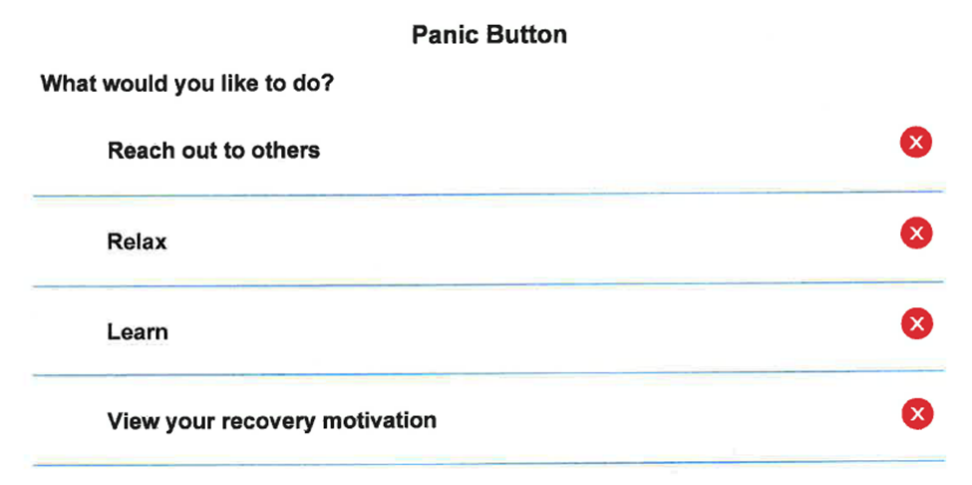
A-CHESS panic button options.

**Figure 3:**
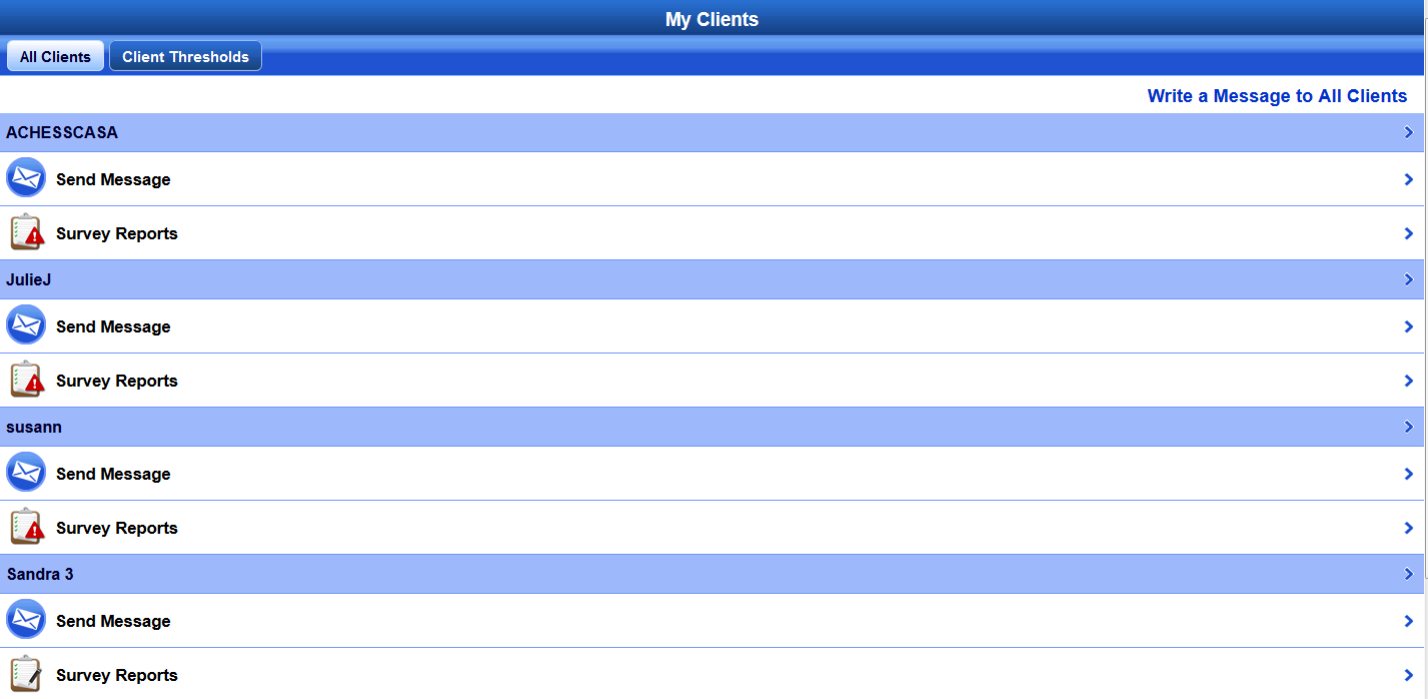
Screenshot of A-CHESS counselor home screen.

A-CHESS assists an individual in recovery through services in the following categories: (1) connection and communication with peers via personal profiles and “walls”, discussion groups, and private messages, (2) assessment of relapse risks through daily check-ins, weekly surveys, and targeted feedback, (3) alerts and reminders to encourage sustained recovery, (4) addiction-related educational materials and recovery news, and (5) tools to manage stress and risky situations that could jeopardize recovery. Figure 1 shows images of the A-CHESS home screen, and Figure 2 shows the Panic Button options. Counselors use an A-CHESS tool to access information on how their clients are doing and connect with them via A-CHESS private messaging. Figure 3 shows a visual of the counselor home screen. In addition, counselors participate in discussion groups and are part of the support team for each individual.

The A-CHESS mobile phone app has been previously tested in a randomized trial involving 349 study participants and found to reduce risky drinking days and improve abstinence [19]. However, a clinical trial provides supports that are not available in most addiction treatment settings. Even though the intervention was efficacious, the question of whether such a technology could be implemented and sustained in a standard clinical practice remains unanswered.

While different frameworks exist to identify facilitators and barriers related to the implementation of organizational change, sustainability of organizational change is an emerging area of research. Existing sustainability frameworks suggest that sustainability is attributable to the (1) internal organizational supports, (2) contextual influences of the external environment, and (3) characteristics of the intervention [31-34]. Innovation characteristics include stakeholder relationships, innovation integrity, effectiveness, and quality of initial implementation efforts, and alignment between innovation and stakeholder (eg, agency staff or patients) needs. Internal organizational supports include champion roles, leadership, resources, policies and procedures, and expert support (ie, coaching). The environment context explores the effects of financial, regulatory, and policy changes. Rather than using a specific sustainability framework (eg, Conceptual Framework for Sustainability of Public Health Programs or Dynamic Sustainability Framework) [31-34], we examined the different elements from each framework to identify innovation, internal organizational, and external environmental factors influencing A-CHESS sustainability. The study sought to distinguish the different strategies employed between agencies that were sustainers and non-sustainers of A-CHESS and also to identify challenges associated with sustained use of A-CHESS beyond grant-funded randomized controlled trials or pilots.

## Methods

### Setting

Agencies involved in this study were all members of the CHESS Health Education Consortium. The consortium brought together University of Wisconsin-Madison researchers and health care organizations to develop and research new health information technologies developed at the UW-Madison Center for Health Enhancement Systems Studies. The focus of the Consortium was to learn how to effectively implement A-CHESS in real-world settings. Treatment agencies that expressed interest in using A-CHESS in their clinics were notified of the opportunity to participate in the research Consortium. Notification took place via phone calls, emails, and at a conference for substance treatment professionals. As part of their Consortium membership, treatment agencies made a donation to the UW-Madison that funded Consortium activities. They also agreed to participate in studies that were generated from the interests of Consortium members. The consortium started in April 2011 and ended in December 2014.

The member agencies made the decision on how A-CHESS would be used within their organizations (eg, what client population, how many participants, what level of care). Members of the Consortium shared information on problems and best practices when implementing and using A-CHESS. The UW-Madison team adapted A-CHESS and expanded administrative tools to help with its implementation and utilization in the different treatment agencies. For example, agencies could add content, develop surveys, customize the meeting locator, or set up a drug testing schedule. The team also revised the use reports to improve ease to use by staff.

### Interview Process

Organizations participating in the Consortium were interviewed at baseline using questions adapted from a previous study conducted with a drug court program using A-CHESS. The previous study was primarily interested in implementing rather than sustaining A-CHESS. The revised interview guide adapted the questions to focus on sustainable implementation and new questions were added to capture processes that would enhance sustainability (available on request from first author). Counselors using A-CHESS with clients were interviewed regarding their use of the app, their perceptions regarding any changes in workload, or how the app had/had not changed their interactions with clients. Program managers were asked about counselor attitudes, workflows, and supervision. The staff interview was repeated at 12 months and 24 months into the implementation process, using some of the questions from the baseline survey. These interviews also asked about organizational and innovation attributes (eg, ease of use or components that users like) that have been found in previous studies to affect sustainability of innovations [31-34]. CEOs/administrators of participating organizations were also interviewed. The baseline interview asked about cost, population, implementation, and thoughts on A-CHESS. The 12-month and 24-month interviews focused on environmental, organizational, and innovation attributes that might have influenced the sustainability of A-CHESS.

### Recruitment

Agency senior leaders were informed of the study at the time they agreed to participate in the consortium. UW-Madison research staff contacted them via email to invite them to participate in an interview and also to identify other agency staff to interview about the A-CHESS implementation. The agency staff was sent separate individual emails that explained the focus of the interview, amount of time required, confidentiality issues, and that participation was voluntary. Consent with study participants was conducted at the start of the initial interview by the UW-Madison research study staff. The study received human subjects’ approval from the UW-Madison Social and Behavioral Sciences Institutional Review Board (SE 2011-0568).

### Data Collection

We conducted 36 baseline and 37 follow-up interviews of staff (counselors and administrators) from 14 agencies. Two UW staff (SD and KP) scheduled and conducted the interviews. Once completed, the interviews were de-identified to remove information that might indicate either a staff member’s identity or the agency identity.

### Definition of Sustainability

While multiple individuals were interviewed within a treatment provider, the presence or absence of strategies to support A-CHESS sustainability were summarized at the organizational level. The primary outcome measure is to assess short-term sustainability, which is defined as whether the agency continued to use A-CHESS (or another mHealth intervention) after the end of the Consortium in December 2014.

### Analytical Approach

The coding scheme was constructed based on theories and frameworks related to both implementation and sustainability [33-35]. We also considered a list of a priori codes that emerged based on how the interview questions were constructed during the preliminary analyses of the dataset. The objective was to capture essential aspects that might influence or prevent sustainable implementation of A-CHESS. After finalizing the coding scheme, we piloted it on a subset of data and refined it through discussions among the researchers into nine main analytic themes with 42 codes nested within themes. Table 1 represents the full analytic framework used in our analysis.

To manage and analyze the blinded interview data, we used Multiuser NVivo, a qualitative data analysis software developed by QSR International. NVivo has components that allow multiple researchers to work collaboratively on data corpus. We utilized the “tree nodes” in NVivo as a structured, hierarchical placeholder for the coding scheme. These tree nodes were then issued to related sections of the dataset allowing the users to run queries on specific codes of interest. We used “annotations” and “memos” to mark the content that required further exploration from coders. These initialed notes were especially helpful for communicating ideas to other collaborators, as they were visible to anyone with access to the document.

A team of 4 researchers collaborated in coding the dataset in NVivo. Each researcher was assigned to a set that included the initial, 12-month, and 24-month interviews. We first completed coding 10% of the data and created comparison reports among coders using “coding comparison query”, a function included in the software [36]. The interrater reliability among coders was 92%. We then allocated the remaining interviews among coders to proceed with the analysis.

The analysis focused on the short-term sustainability of A-CHESS at the organizational level. Input from staff and leadership provided different perspectives about the strategies employed. The interview guides asked similar questions of both staff and leadership, with the primary difference being that leadership was asked additional questions about how the external environment (eg, regulations or funding) might influence A-CHESS sustainability. The baseline interviews and follow-up interviews as well as interviews by staff type were initially analyzed separately to explore themes related to the implementation and sustainability of A-CHESS. However, as we moved further with our analysis, we found that the line between implementation and sustainability became blurred as many of our codes (such as “monitoring of sustainability” and “implementation barriers”), as well as the opinions of staff and leadership, overlapped. We realized that a complete picture of the organizational strategies employed to support A-CHESS sustainability could not be achieved without integrating the results into a holistic picture for the organization. Therefore, we did not draw a sharp line between the definitions of implementation and sustainability and did not distinguish between responses from staff and leadership.

**Table 1:** A-CHESS coding scheme.

Domain	Code		Description
Physical structure	Age		Age of the interviewee
	Gender		Gender of the interviewee
	Employment status		Administrator or staff, full time or part time.
	Weekly scheduled shifts		Hours of weekly scheduled shifts, including weekly night shifts and extra shifts.
	Client population		Any mention of client population, clients served with A-CHESS, any other characteristic that describes client norms, trends, or patterns, number of clients.
Communication	Method of communication		Any mention of how they communicate with clients, email, phone, text messaging, and preferred method of communication.
	Hours spent communicating		Any mention of hours spent communicating with clients, hours spent with one client, off-duty hours spent with clients.
	Client awareness		Any mention of knowledge of clients’ health welfare, availability of emergency care, and clients’ support mechanisms.
Readiness for A-CHESS	Access to technology		Any mention of technology they use regularly, use of electronic records, and access to Internet.
	Prior training		Any mention of attendance to webinars/conferences, whether they have continued training, whether it was sufficient, whether there were any helpful mediums, suggestions.
	Assumed benefits		Any mention of benefits of using A-CHESS with clients prior to the implementation of the tool.
	Reactions		Any mention of reactions, statements about learning curve prior to the implementation of the tool.
A-CHESS implementation	Implementation barriers	Technical barriers	Any mention of applications not working, difficult to use/understand applications.
		Resistance	Any mention of barriers to implementation such as staff resistance, leadership resistance, client resistance
		Insufficient funding	Any mention of payer restrictions, expenses, or cost indicated as implementation barrier.
		Other factors	Any mention of regulations that directly or indirectly limit access or any other internal or external factors that hinder implementation.
	Overcoming barriers		Any mention of how barriers were overcome, methods supporting the implementation.
	Improvements		Any mention of suggestions, improvements, whether they would recommend it to others.
	Useful applications		Any mention of applications they used most, applications they liked, applications that are easy to understand, and comments on the helpfulness of applications.
	Less useful applications		Any mention of applications they felt like they did not need, disliked.
A-CHESS involvement	Involvement with A-CHESS		Any mention of devices used with A-CHESS, time spent on implementation, A-CHESS use per shift, A-CHESS use off work.
A-CHESS impact	Positive internal impact		Any mention of positive impact of A-CHESS use on client relationship, on workload, daily activities.
	Negative internal impact		Any mention of negative impact of A-CHESS use on client relationship, on workload, daily activities.
	Positive external impact		Any mention of state and/or federal regulations or policy (including Affordable Care Act) that positively impact use of A-CHESS.
	Negative external impact		Any mention of state and/or federal regulations or policy (including Affordable Care Act) that positively impact use of A-CHESS.
Sustainability	Strategies employed		Any mention of strategies employed for sustainability including strategies to overcome any barriers. Note: When coding, name the strategies employed.
	Monitoring of sustainability		Systems in place: Any mention of processes implemented to routinely measure the impact of A-CHESS on client or organizational outcomes
			Communication: Any mention that A-CHESS impact is shared with staff, leadership
			Frequency: Any mention of the frequency with which A-CHESS results are shared
	Outside environment		Any mention positive or negative of how outside influences, such as regulatory changes or absence of funding to support efforts to bill for technology use, impact sustainability.
	Challenges to sustainability		Any mention of challenges that inhibit sustainability, including staff resistance to A-CHESS. Note: Enter notes about the different barriers or challenges
	Attributes enhancing sustainability		Any mention of A-CHESS attributes enhancing sustainability.
	Attributes inhibiting sustainability		Any mention of A-CHESS attributes inhibiting sustainability. Note: attributes from the client perspective should be also be coded with client population.
	Resources to support sustainability		Any mention of resources (such as staff and funding) needed to sustain changes and how they will be obtained, and how sustainability will be measured. Note: When coding, we should note which strategies were employed.
	Staff engagement		Any mention positive or negative of how staff was engaged in ongoing efforts to sustain A-CHESS.
	Financial impact		Any mention of the business case for sustaining A-CHESS including whether or not the business case (ie, financial gains) associated with A-CHESS have been successful or not successful.
	Facilitator impact		Any mention of which facilitators turned out to be the most important in sustaining A-CHESS within the organization.
Funding	Funding		Any mention of funding or cost, including funding resources to support sustainability, regulations affecting funding or cost, the business case for continuing A-CHESS, expenses, and compensations.
Other technologies	Technology type		Any mention of a new technology that the organization will be or has implemented for their clients.
	Technology platform		Any mention of the platform for which the new technology needs to operate.

## Results

### Overview

Detailed information about the organizational attributes of the A-CHESS consortium agencies is available in Multimedia Appendix 1. The 14 agencies are geographically distributed equally across the country except for the West region (n=2). The majority of the agencies (9/14, 64%) serve more than 500 clients per year. Table 2 provides an overview of the interview process by agency, including staff interviewed and dates, as well as the date the agency joined the consortium.

A total of 73 interviews were completed with 44 unique individuals. Interviews were conducted with staff (n=24) and leadership (n=20) across the 14 agencies. Table 3 shows that the majority of staff interviewed was female (14/24, 58%); however, 60% (6/10) administrators were male. The staff workforce reported an average tenure of 5.7 years with 46% (6/13) being with their agency less than 3 years. Also, 13 out of the 24 staff (54.2%) reported their age was under 40. Smartphone use was prevalent among the staff members under age 40.

**Table 2:** Interview timeline by agency.

Agency	Consortium join date	Roles	Initial interview date	12-month interview date	24-month interview date
1	10/1/2011	Admin	6/25/2012	7/23/2013	8/12/2014
		Admin	None	7/10/2013	Several contacts, no response
		Staff	None	8/20/2013	8/25/2014
		Staff	6/26/2012	None	N/A
2	4/1/2011	Admin	2/20/2012	None	N/A
		Admin	4/11/2012	7/23/2013	8/5/2014
		Staff	4/10/2012	7/25/2013	8/4/2014
		Staff	4/10/2012	None	N/A
		Staff	None	7/17/2013	7/30/2014
3	11/1/2011	Admin	None	7/24/2013	7/25/2014
		Staff	6/25/2012	7/24/2013	4 reschedules
		Staff	None	7/16/2013	7/24/2014
		Staff	7/15/2013	None	N/A
4	1/1/2013	Admin	5/1/2014	5/1/2014	
		Admin	6/13/2014	6/13/2014	
5	4/1/2011	Admin	2/29/2012	8/2/2013	Several contacts, no response
		Staff	2/6/2012 (2 staff together)	None	N/A
		Staff		None	N/A
		Staff	None	7/30/2013	8/21/2014
6	11/1/2011	Admin/ Staff	7/5/2012 (admin interview)	7/31/2013 (staff interview)	8/29/2014
		Admin	3/2/2012	None	N/A
		Staff	None	9/6/2013	Not in agency anymore
		Staff	8/7/2012	None	N/A
7	11/1/2011	Admin	6/25/2012	7/30/2013	8/13/2014
		Admin	6/26/2012	7/10/2013	8/12/2014
		Admin	6/26/2012	7/11/2013	not in agency anymore
		Staff	6/21/2012	7/25/2013	Several contacts, no response
		Staff	7/11/2012	None	N/A
8	7/1/2011	Admin	3/5/2014 (24 mos.)	Not in consortium	
		Staff	3/5/2014 (24 mos.)	Not in consortium	
9	4/1/2011	Admin	2/7/2012	Not in consortium	Not in consortium
10	7/1/2011	Admin	6/7/2012	Not in consortium	Not in consortium
11	1/1/2013	Admin	10/28/2013	N/A	
		Staff	11/1/2013	10/23/2014	
12	7/1/2013	Admin	1/15/2014		
		Staff	1/17/2014	1/16/2015	
		Staff	1/28/2014	1/26/2015	
13	11/1/2013	Admin	1/24/2014		
		Admin	2/7/2014	2/25/2015	
		Staff	1/22/2014	1/12/2015	
		Staff	1/27/2014	1/23/2015	
14	5/29/2013	Admin	5/6/2014		
		Staff	5/29/2014	5/29/2014	

**Table 3:** Qualitative inquiry response rate and respondent demographics.

			Staff	Administrator
**Interview time frame, n**				
		Initial interview	17	19
		12-month interview	15	11
		24-month interview	5	6
**Employee demographics (based on responses received), n (%)**				
	**Gender**			
		Male	10 (41.7)	12 (60.0)
		Female	14 (58.3)	8 (40.0)
	**Age range**			
		20-29	5 (20.8)	
		30-39	8 (33.3)	
		40-49	3 (12.5)	
		50-54	2 (8.3)	
	Refused/no response		6 (25.0)	
	**Access/use of technology**			
		Computers	22 (47.8)	
		Mobile phone/smartphone	19 (41.3)	
		iPad/iPod	4 (8.7)	
		Other (videophone)	1 (2.2)	
	**Tenure in years**			
		˂3 years	6 (46.1)	
		4-9 years	5 (38.5)	
		˃9 years	2 (15.4)	
	Average tenure in years, mean (SD)		5.7 (5.2)	

### Qualitative Analysis

Identification of agencies meeting our definition of short-term sustainability was straightforward. Three agencies (#5, #7, and #13) continued to utilize A-CHESS as an integrated part of their service delivery after the Consortium ended. Three agencies (#8, #9, and #10) dropped out of the Consortium before the 12-month interview. Although the remaining agencies were not able to sustain A-CHESS after the Consortium ended, their efforts were also reviewed to help identify unsuccessful sustainability strategies. We utilized the previously identified sustainability frameworks to organize the qualitative analysis which suggest that sustainability is attributable to the internal organizational supports, the external environment (eg, funding and regulations), and characteristics of the intervention [31-34]. Our analysis focused on (1) the internal organizational supports put in place to sustain A-CHESS, (2) funding as a contextual influencer from the external environment, and (3) A-CHESS innovation characteristics that support its sustainability.

### Internal Organizational Supports

Organizational supports focused on three specific strategies: leadership support, staff engagement, and client engagement. Strong leadership support was present across multiple agencies. Often in these agencies, the leadership team took systematic and organized steps to move forward with the program. One respondent stated, “The whole system had to be developed from the ground up including process development and documentation”. In another agency that sustained A-CHESS, leadership understood that using technology to engage clients in treatment is innovative. However, the external environment (eg, reimbursement from insurance providers or Medicaid) is not keeping up with the innovations. As a result, leadership in this agency recognized that external funding to support A-CHESS would need to come from private donors and/or foundations. The absence of leadership support may hinder development of an A-CHESS business model strategy. A respondent from one agency not sustaining A-CHESS stated that they need to be “able to convince administrators that there is a financial benefit to them”.

### Staff Engagement

Engaging staff during the initial implementation and ongoing support of an mHealth app like A-CHESS is an important attribute associated with sustainability. The three agencies that were able to sustain A-CHESS used three clearly defined concrete strategies to engage staff (Table 4). One strategy involved the regular review of reports about client use of A-CHESS. This provided staff with additional opportunities to identify clients at risk and to initiate follow-up efforts to re-engage them. One respondent highlighted the importance of monitoring from a clients’ perspective when they stated the value of the relapse prevention feature (weekly check-in): “Clients like [A-CHESS], it’s a small and valuable intervention”. Another staff engagement strategy involved integrating a discussion of A-CHESS into weekly staff meetings. One respondent indicated that “Once a week we sit down and talk and review globally their caseload, [to] extend utilization of A-CHESS”. The final staff engagement strategy these three agencies used involved identifying staff who expressed an interest in working with A-CHESS. For example, one respondent indicated that they “selected staff that expressed interest so they’re invested so we don’t have any staff resistance”. A response from one of the agencies highlighted the integration of dedicated staff with the weekly review process when they stated, “We have a team of folks who focuses only on A-CHESS, assess on how clients are using A-CHESS. We have dedicated staff and we monitor client outcome”. Other strategies such as providing resources, using peers to provide training, or producing A-CHESS guides were specific to only one of the agencies; while a few of the non-sustaining agencies (#1 and #6) employed one of these key strategies. For example, Agency 1 utilized the A-CHESS reports to provide information to staff about client usage; however, unlike Agencies 5, 7, and 13, that information was not shared with staff on a regular basis. A respondent from one of the agencies that successfully sustained the use of A-CHESS summarized the impact and importance of staff engagement by stating that “we work with patients more at the beginning to sustain interest, help them use the phone…[and]…developed some guidelines”. These changes helped patients learn more about how the phone app works. In turn, these individuals were able to help new patients who were not familiar with the phone by providing hands-on support through peer support meetings.

**Table 4:** Organizational and external environmental strategies supporting sustainability.

			Agency													
			1	2	3	4	5	6	7	8	9	10	11	12	13	14
**Organizational level strategies**																
	Strong leadership support and engagement		√	√			√	√	√					√	√	√
	**Staff engagement**															
		Implement reminders related to A-CHESS use for staff	√													
		Provide and communicate information through regular review of reports on client usage	√				√		√						√	
		Offer training to new staff and ongoing training to all staff including face-to-face meetings to promote staff mentoring opportunities		√		√		√								
		Require new staff to be excited and familiar with technology											√			
		Produce A-CHESS booklets with screenshots customized to the treatment location or organization specific A-CHESS program guides				√			√							
		Establish a web link for A-CHESS demonstrations and video tutorials				√										
		Identify staff interest in working with A-CHESS					√		√						√	
		Incorporate discussion of A-CHESS into weekly staff meetings					√	√	√						√	
		Involve peers in training							√							
	**Ongoing client engagement**															
		Use feedback reports to identify clients not using the system and have staff follow-up	√				√		√						√	
		Establish guidelines and contracts related to use of A-CHESS or discharge protocols about how to use A-CHESS	√				√		√							
		Use peer counselors or clients to provide training on how to use A-CHESS or share their experiences							√						√	
		Adopt A-CHESS materials for specific client needs		√												
		Encourage client use of A-CHESS features such as discussion group				√										
		Establish task force or internal work group of internal champions including clients to engaged clients in A-CHESS					√		√						√	
		Create training sessions for clients including developing user manuals						√	√							
		Engage clients early on by showing them how to use the phone							√							
		Establish targeted marketing for selected clients		√	√				√					√		
	**General or generic**															
		Increase public relations by going to community based gatherings		√												
		Used in the agency to augment or replace existing therapeutic approaches				√										
		Provide resource supports such as office space and staffing support					√								√	
		Work to establish or increase stakeholder awareness of A-CHESS and its benefits				√			√						√	
**External environment financial business model**																
		Leverage billing codes to support reimbursement for A-CHESS													√	
		Seek out other sources of funding (eg, donations, mini-grants, etc)			√		√		√						√	
		Communicate with insurance carriers or other external agencies to drive support and seek funding													√	
		Work with phone carriers to see if lower rates can be secured													√	
		Developed a business case for A-CHESS service line					√		√							

### Ongoing Client Engagement

Client engagement strategies can promote initial client engagement or ongoing engagement once the client starts to use the technology. While both are important, sustainability relies more on the identification of strategies that encourage ongoing client use of the intervention. Each of the agencies that sustained A-CHESS used two specific strategies to promote client engagement. The first strategy focused on monitoring client engagement with A-CHESS. Staff in these agencies utilized the available reports to identify clients who were not using the system and created processes that required follow-up with the client. As one respondent indicated, “It is up to the staff to engage the people. Peer to peer is not so high so we do need our staff to engage them. Staff participation is very important”. In contrast, staff at agencies who did not sustain A-CHESS identified a lack of communication as a barrier to sustainability but did not have a solution to systematize the feedback process or had to determine if these reports were a better source of data than internal tracking systems. As one staff member responded, “The Excel spreadsheet does not give you much unless you know what data to analyze, what variables to use”. In some agencies that did not sustain A-CHESS, other organizational changes such as system upgrades or electronic medical record implementation affected staff time to successfully manage the data from A-CHESS.

The second client engagement strategy focused on efforts to establish an agency team (eg, a task force or internal work group) consisting of dedicated staff (and in some instances, clients), whose responsibility was to help promote client engagement with A-CHESS. One agency created a task force of internal champions whose primary role was “all about staying connected to the clients”. As a result, staff indicated that A-CHESS helped improve their interactions with clients. A staff member in one of these agencies stated, “It has made my work easier as I can work with patients over the phone and they do not have to come in as often”. Another staff member defined ACHESS as “a game-changing event”. In this agency, leadership considered “enthusiasm for the program [A-CHESS]” when making counselor hiring decisions.

Other client engagement strategies were used by some but not all of the agencies sustaining A-CHESS. Two of the agencies (#5 and #7) also established protocols, guidelines, or contracts designed to define client expectations for using A-CHESS while in treatment. A respondent from one agency stated that “a client agrees to expectations outlined in a document which he/she signs”. The protocols also help clients remain engaged with A-CHESS upon discharge from treatment. Another respondent from a different agency stated, “We offer A-CHESS to our patients in the 28-day inpatient program. It is part of the discharge plan. We talk about it in our weekly alumni group. Before they leave, we give them their phone back (to those who have phones before checking into our inpatient program) and meet with them, put A-CHESS in their phone so they are all set before they walk out of the door”. Agency 1 also used this strategy and the feedback reports but did not sustain A-CHESS after the Consortium ended.

In addition, two agencies (#7 and #13) used peer counselors or staff to train clients on A-CHESS use. A respondent from one of these agencies stated, “Peer mentoring staff and clinical staff heavily promote A-CHESS utilization as an important addition to quality and continuum of care”. Staff in Agency 7 expanded on these efforts by making sure that clients were trained in how to use A-CHESS. Specifically, staff and peers provided training when the client was first given the phone. While other strategies were applied to promote ongoing client engagement, agencies that sustained A-CHESS did not use them.

### External Environment: Financial Business Model

Developing a business model to support the ongoing use of mHealth apps (eg, A-CHESS) is a key strategy for long-term sustainability. Agencies that sustained A-CHESS (#5, #7, and #13) took active steps to reduce costs (eg, provided to clients with a phone) and explored the possibility of additional funding. Leadership in these three agencies used a variety of different strategies to seek or secure additional funding (Table 4). For example, all three agencies sought other sources of funding such as mini-grants or donations. In addition, Agency 13 took advantage of state Medicaid billing codes related to peer monitoring, case management, and crisis intervention to support A-CHESS. This agency also worked closely with the MCOs in the state who “are engaged in A-CHESS promotion and are devoting funds to ‘pilot projects’ for A-CHESS implementation with their patient population”. Two agencies (#5 and 7) that have sustained A-CHESS have taken a different approach to incorporating A-CHESS into their business model. For example, Agency 5 established an A-CHESS service line and developed presentations about A-CHESS impact on client outcomes. Agency leadership uses the presentations to communicate their message to potential donors or foundations, which has helped brand the agency as a leader in the field. They had this to say about their approach: “We’ve incorporated technology in our service line and we’re staying on it. In presentations, we promote and talk about A-CHESS…when I do the presentation and show A-CHESS, I get the audience’s attention”. As part of the business model strategy for this agency, A-CHESS was offered only to clients with a smartphone and associated data plan, thus avoiding the variable expense associated with buying phones and providing the data service plan.

### Absence of a Financial Business Model

Agencies that did not sustain A-CHESS recognized the importance of securing outside funding to support ongoing use of the app. Leadership in three of those agencies (#1, #2, and #11) took active steps to reduce costs and explored the possibility of additional funding including switching carriers, initiating conversations with the state about Medicaid coverage, and using A-CHESS reports to highlight the effectiveness of A-CHESS to the community. Despite these steps, one respondent from these agencies stated, “Funding is always the top barrier. We’re always looking for ways to support the A-CHESS program”. Another respondent indicated that one of the challenges to sustaining A-CHESS was the “cost of phones; clients aren’t really using [A-CHESS] that much the longer they are in treatment”. Agencies that relied on grant funding to pay for staff in the A-CHESS pilot were struggling with figuring out how to continue to pay for those staff in the future. Other agencies that did not sustain A-CHESS did not appear to implement any of the successful business model strategies to help cover costs or secure additional funding. In one of these agencies, the absence of a business strategy combined with financial instability indicated that more work was required “to integrate [A-CHESS] into existing treatment system”.

### A-CHESS Innovation Characteristics

Attributes of an innovation influence whether it is sustained within an organization. Table 5 summarizes innovation enhancers and inhibitors across all Consortium agencies about specific A-CHESS attributes might contribute to its sustained use their clients.

The top three innovation enhancers mentioned by staff were the discussion group, the ability of A-CHESS to address client needs and staff support, and rapid response to client concerns. Staff believed that A-CHESS allowed the clients to create a sense of community in order to help each other in a time of need. One respondent stated that using A-CHESS “brought all clients to be closer together, they never met each other before but feel connected, they call themselves ‘Band of Brothers’”. Another respondent said, “It’s exciting that technology is part of recovery. It’s mobile, on the run, you just press 2 buttons and you can get in touch with people. It allows the client to reach out anytime”. Even agencies that were not able to sustain A-CHESS still recognized the importance of engaging clients. Staff in one agency encourages clients to “share in Discussion Group, like when they share in a 12-step meeting…[because it is] Secure, it’s safe. It’s the recovery version of Facebook. They can communicate and know they are being monitored”. A respondent from another agency indicated that A-CHESS “helps them [clients] make appointments, keep up with appointments, [and] continue with their therapy”. Despite recognizing how A-CHESS helped with client engagement, these non-sustaining treatment agencies did not actively develop and implement strategies to promote client engagement. The reason identified in one of these agencies was “the failure to put time and work with the clients who didn’t use A-CHESS”.

**Table 5:** Attributes of the A-CHESS innovation potentially impacting sustainability.

		Agency													
		1	2	3	4	5	6	7	8	9	10	11	12	13	14
**Innovation enhancers**															
	Discussion group		√	√		√	√	√					√	√	√
	Addresses client need (eg, rides, schedules, or information)	√	√			√	√	√					√		√
	Staff support and rapid response (eg, BAM survey or panic button)	√	√		√		√	√						√	√
	Panic button		√		√	√		√							
	Ease of use	√	√	√				√							√
	Communication with peers				√								√	√	
	Impact on staff workload (eg, makes their jobs easier)	√	√												
	Thought of the day		√												√
	Weekly survey													√	
	My profile	√													
**Innovation inhibitors**															
	Availability and cost of phones	√	√										√	√	
	Concerns about being tracked			√										√	
	Technological failures (eg, no coverage, features not working etc)	√	√	√			√								
	Clients skills or comfort level with smartphones, which may impact regular use of the phone	√					√								

### Technology

Technological challenges, phone availability and costs, concerns about being tracked, and the skills of the users were identified as potential innovation inhibitors. During the initial rollout, the phone or features on the phone (eg, meeting locator) did not always work as expected for both clients and staff. One respondent stated, “There are a lot of problems with phones (not good quality). We thought at first it was the clients not taking care of the phones but staff also had problems with phones”. Another respondent indicated that “it’s too long to load, sometimes get a blank screen… but some clients have complained about not getting signal (connectivity)”. These challenges affected clients’ use of the phone. For example, one respondent stated, “It is a problem because clients cannot call for transportation”. Client comfort level with technology in general could also be a barrier to sustained use. One respondent indicated that “some of the older guys are not tech savvy; we have to teach them over and over again, tell them that they can play with the phone and it won’t hurt it”. Finally, client concerns about the tracking function of the innovation were raised in a couple of agencies. One respondent stated, “The paranoia effect, clients think we’re tracking them and following them with electronic capability”.

## Discussion

### Principal Findings

Strategies that might influence sustainability may be related to intervention types [37]. A-CHESS is an innovation that requires coordinating multiple staff to implement a new technology in the provider organization. Efforts to successfully sustain such an intervention require support from leadership and a project champion, an understanding of how the innovation fits within the organizational mission, training for all staff involved in the implementation, seeking continued financial resources, integrating a new practice into organization policies and procedures, and continuous monitoring [37]. Our results indicate how these strategies help sustain the use of mHealth apps within the context of addiction treatment programs.

While leadership support is an important part of the different sustainability frameworks [31-34], how it unfolds in the organization is less clear. We found that many agencies indicated the presence of leadership support for A-CHESS implementation. Involved and committed leaders can help facilitate sustainability [38-40]. In agencies that successfully sustained A-CHESS, leadership was actively involved by providing support during implementation. These leaders took steps to ensure that their strategic decisions and plans for A-CHESS created a coherent vision for organizational transformation; empowered employees to participate in the change process, including providing training; and allocated resources, change policies, procedures, and job descriptions [31,41,42]. For example, leadership in these agencies recognized the need to establish A-CHESS use covenants or contracts before providing the innovation to a client.

Fiduciary responsibility is a leadership task. Efforts to secure ongoing funding are an important issue in mHealth implementation [25,43]. Our results indicate that leadership can support efforts for mHealth financing by establishing service lines, leveraging billing codes, and marketing the impact of mHealth on clients to perspective payers. Future research should explore how these different approaches contribute to the long-term sustainability of mHealth apps.

Effectively engaging staff, and in our case, clients, establishes a sense of empowerment that they are a part of the change process, enhances staff motivation to participate in the change process, and leads to more actionable steps by staff to promote sustainment of the change [40,44-48]. We found that specific strategies targeting staff and client engagement differed between agencies that sustained versus did not sustain use of A-CHESS. For example, successful agencies established A-CHESS implementation teams and utilized clients and peer counselors to train new users. As a result, the participation helped to provide sufficient evidence for staff and clients to believe in the benefits of A-CHESS. This local participatory approach enhances sustainability efforts [39,49-51]. Further research is required to understand how staff and client engagement strategies support the sustainment of mHealth apps.

Developing an ongoing measurement and feedback system facilitates and supports organizational sustainability efforts [39,50,52-54]. It has been suggested that such systems have four components: (1) continually and effectively monitor progress of change, (2) keep the organization informed about success, (3) identify new areas for improvement, and (4) establish protocols to communicate and act on the results [55]. Agencies sustaining A-CHESS developed systems focusing on three of the four components, excluding a focus on new areas for improvement. Their strategies integrated monitoring efforts into weekly meetings and established policies and procedures to follow up with unengaged clients. The effectiveness of the approach on client engagement requires further investigation.

### Limitations

Providers that participated in the research consortium (a self-selected sample) may not be representative of other treatment providers that might have implemented other mHealth apps. Another limitation related to the sample is that these providers implemented a specific mHealth app—a recovery-oriented app for individuals with substance abuse issues. The strategies associated with implementing and sustaining the A-CHESS app may differ from other mHealth apps. For example, levels of direct care staff engagement associated with use of another mHealth app may require different strategies to promote sustainability.

The qualitative process relied on a sample of interviews from the participating agencies. Staff turnover meant that different staff members were interviewed for the baseline and follow-up surveys. Inconsistency in the timing of the interview is another study limitation. The interviews did not begin when an agency joined the consortium. Time was required to allow the agency to develop a plan to implement A-CHESS in their organization. As such, the initial interview was not conducted until after the plans were developed with a range of 3-16 months between when the organization joined the consortium and when we conducted the initial interview. We then attempted to conduct the follow-up interviews at the appropriate 12-month and 24-month windows. Efforts to schedule the interviews with staff led to, in some instances, the interviews being scheduled as much as 1-5 months outside of the respective 12-month and 24-month timeframe. In addition, the interview process did not include patients using A-CHESS. Their responses about strategies to sustain A-CHESS may have differed from staff. Client perspectives about why they continue to use a specific mHealth technology should be included in future studies.

The external funding strategies are specific to the structure and financing mechanism within the US health care system for substance abuse treatment. The organizational strategies might be transferable to non–US-based substance abuse treatment providers or other types of health care systems. However, the full implementation of the Affordable Care Act in the United States or the actual structure of the payment system in another country may require changes to existing strategies or the identification of new strategies for sustaining an mHealth app in general or A-CHESS in particular within their respective implementation environments. Further research will be necessary to fully understand how different financial strategies and payment mechanism affect the use of mHealth apps in health care.

### Conclusions

As with any technology adoption, the use of mHealth apps for disease management will require a series of changes to the operations in health care provider organizations. Successful organizations will develop adoption plans that address staff and patient engagement, organizational procedures, and business model. Organization leaders will revisit these plans and adapt their implementation process to address emerging needs as they arise. Future research should focus on understanding the environmental, organizational, and innovation attributes that influence an organization’s decision to sustain the use of a particular mHealth app for its clients.

## Appendix

Multimedia Appendix 1 A-CHESS consortium member organizational attributes.

## Abbreviations

A-CHESS: Alcohol Comprehensive Health Enhancement Support System
BAM: Brief Alcohol Monitor
