# Supplementary material for: Successful Organizational Strategies to Sustain Use of A-CHESS: A Mobile Intervention for Individuals With Alcohol Use Disorders
Source: J Med Internet Res. 2015 Aug 18;17(8):e201. doi: 10.2196/jmir.3965 (PMC4642385; doi:10.2196/jmir.3965)
Supplement: Multimedia Appendix 1 [file jmir_v17i8e201_app1.pdf]

Multimedia Appendix 1. A-CHESS consortium member organizational attributes.

| Agency | Sustained A-CHESS | # of Admissions | # of FTE's | Geographic Location | Client population using A-CHESS |
|---|---|---|---|---|---|
| 1 | No | 12,000 outpatient, emergency, and community justice programs. 56 served annually in substance abuse programs | 1000 employees (overall); 6 FTE in SA programs | Northeast | Drug court population, 18 years and older |
| 2 | No | Fiscal year 2013: 13k clients, and among them, there were 25719 admissions. This means some clients had multiple admissions per year. | 343 staff members at 13 locations | West | Deaf, hard of hearing adults |
| 3 | No | Calendar year 2013 (excluding those clients served in CD Access & Engage, Federal Probation - Collection Only, and Adult Detox) 1,483 unduplicated clients in its various adult CD programs. | Employs approximately 300 individuals; 25 in CD programs. | Midwest | Adults with substance abuse and mental health issues. |
| 4 | No | Serves up to 500 clients per year (256 clients signed up for A-CHESS). | 10 in agency; 80 therapists in its provider network. (23 staff using A-CHESS) | South | 16 agency partners. These include MAT and correctional groups, as well as community behavioral healthcare, residential and transitional centers. |
| 5 | Yes | Over 2500 patients served per year; 1400 admissions | 264 FTEs | Northeast | Graduates of an in-patient rehabilitation program. |
| 6 | No | Over 2500 patients served per year. (694 Substance Abuse Admissions for Jan 2013-Dec 2013) | 536 providers in 58 sites across all 8 counties. (Clinicians=10, Recovery coach=4, Recovery Manager=7) | South | Females, 18-40, who are pregnant or who could become pregnant. |
| 7 | Yes | Calendar year: 1275 clients served at two locations. | 43 FT employees | Northeast | Primarily Vietnam vets with addiction. |
| 8 | Dropped | In 2012: 27, 469 people | More than 430 | West | Substance |

| | | | | | |
|---|---|---|---|---|---|
| | out of consortium | received clinical services, 13,083 assessments completed, 11,876 received outpatient services. (Over 2500 patients served per year) | full and part-time employees | | abuse with mental health clients |
| 9 | Dropped out of consortium | 1297 admissions in 2013 | 6 FTE counselors in inpatient unit | Midwest | Adults of all ages with co-occurring disorders |
| 10 | Dropped out of consortium | Serves 500-2500 clients per year | Unknown | South | Adults (ages 25-40) leaving residential treatment. |
| 11 | No | Provides over 350 women, men and children with over 100,000 nights of shelters. Transition program provides housing, addiction recovery support, and job training for 18 people | 3 staff in transition program. | Midwest | Homeless (little to no income) with substance abuse issues and at least 30-day sobriety. |
| 12 | No | Close to 5000 admissions in FY2013 + outpatient services. | 143 FTE's | Northeast | Adults with substance abuse, also dual diagnosis |
| 13 | Yes | 895 individuals were admitted into treatment services at four locations.<br>370 admissions in calendar year 2013 | 67 FTE's. | Midwest | Adults with substance abuse |
| 14 | No | Fiscal year 2013: served 183 clients. | 20 to 50 staff; 5.5 FTE in program utilizing A-CHESS | South | Criminal justice and substance abuse clients, treatment provider for drug court. |
